# Neuroborreliosis Presenting as Guillain-Barré Syndrome

**DOI:** 10.7759/cureus.42322

**Published:** 2023-07-23

**Authors:** Jacob Farr, Jan Bittar

**Affiliations:** 1 Department of Neurology, The Ohio State University Wexner Medical Center, Columbus, USA

**Keywords:** guillain-barré syndrome, western blot, albuminocytologic dissociation, ascending paralysis, demyelination, tick, borrelia burgdorferi, guillain-barré syndrome (gbs), lyme disease, neuroborreliosis

## Abstract

Lyme disease (LD) is the most common vector-borne disease in the United States. The early localized disease presents with erythema migrans and nonspecific constitutional symptoms. A neurological manifestation of LD (neuroborreliosis) is only seen in 10-15% of LD cases, and it typically presents as cranial neuritis or painful radiculitis. We report a case of a 33-year-old male who presented with progressive ascending bilateral lower extremities weakness with paresthesia in hands and feet following an upper respiratory tract infection and an abdominal rash. Cerebrospinal fluid (CSF) analysis revealed albuminocytologic dissociation. An electrodiagnostic study showed prolonged distal motor latency, conduction block, and absent F-wave response. Magnetic resonance imaging of the lumbar spine revealed enhancement of the cauda equina nerve roots. After a lack of improvement with intravenous immunoglobulin for presumed Guillain-Barré syndrome (GBS), Lyme serologies were sent and showed positive Lyme antibodies in serum and CSF as well as positive western blot IgM followed by IgG seroconversion a week later. The patient was started on IV ceftriaxone and doxycycline for four weeks with significant improvement in his symptoms. This is a rare case of LD presenting as GBS. Lyme can have diverse neurologic manifestations and should be considered in the differential diagnosis of GBS in the appropriate settings.

## Introduction

Lyme disease (LD) is the most common vector-borne disease in the United States, with an annual report of 476,000 cases since 2010 [1]. These cases are expected to rise by over 20% in the coming decades due to climate change [2]. LD is a bacterial infection caused by a spirochete called *Borrelia burgdorferi* and rarely by *Borrelia mayonii*, and it is transmitted by the bite of infected ticks of the genus *Ixodes *[3]. LD can be divided into three stages: early localized, early disseminated, and late disseminated. Untreated LD can produce a variety of symptoms depending on the stage of infection. These symptoms include erythema migrans, nonspecific constitutional symptoms, arthritis, carditis, and various neurologic manifestations termed as neuroborreliosis [4]. The first case of neurologic Lyme borreliosis was reported by Garin and Bujadoux in 1992 when they described a patient in France with meningoradiculitis after an erythema migrans following a tick bite [5]. Neuroborreliosis is only seen in 10-15% of LD cases, and it most commonly presents as painful radiculitis, cranial palsy (mostly facial palsy), and headache [6]. Peripheral demyelination in neuroborreliosis, as we describe in this case, is exceedingly rare [7]. Here, we report an atypical presentation of neuroborreliosis with clinical, radiographic, and neurodiagnostic findings mimicking Guillain-Barré syndrome (GBS).

## Case presentation

**Figure 1:**
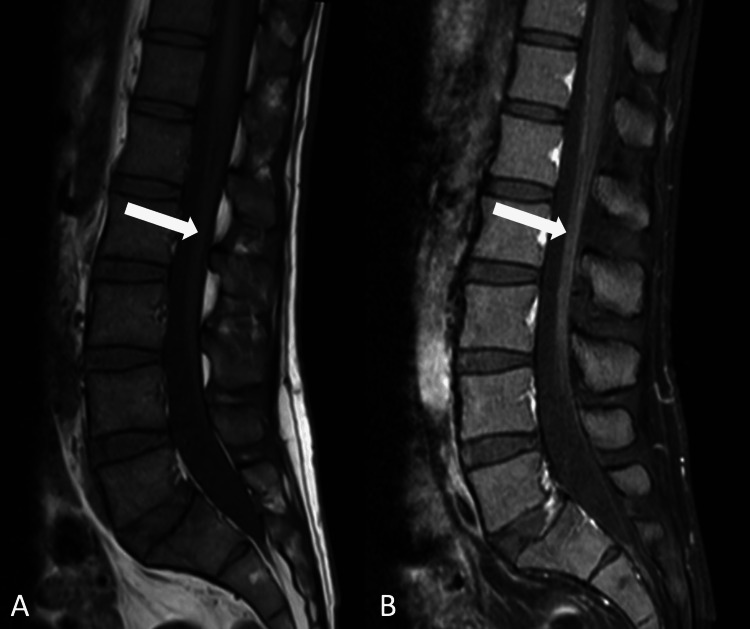
Magnetic resonance scan of the lumbar spine (T1 weighted) Diffuse nerve root enhancement of the cauda equina with mild thickening. A: sagittal pre-contrast; B: sagittal post-contrast.

A previously healthy 33-year-old male with no chronic medical history presented with the progressive onset of bilateral hands and feet numbness associated with an ascending symmetric paraparesis (Medical Research Council (MRC) scale of 4/5 proximally and 3/5 distally). The patient reported that his symptoms started one week ago as a “tingling sensation” in his hands and feet with perioral numbness along with a concurrent fever, nonproductive cough, and a “blotchy” abdominal rash, which was not present upon admission. He denied any recent travel history or tick bite. He works as a 4th-grade teacher and frequently plays disc golf in wooded environments in his hometown in Ohio. The patient was afebrile upon presentation with no cough, chest pain, or shortness of breath. The physical exam also revealed bilateral facial palsy as well as hyporeflexia at his knees and ankles with downgoing plantar responses. The sensation to light touch was decreased below the elbows and up to his knees. Complete blood count (CBC) and basic metabolic panel (BMP) were within normal limits. Chest X-ray did not reveal any acute cardiopulmonary process. He underwent lumbar puncture, and CSF analysis revealed a white blood cell (WBC) count of 0 (range: 0-6 /uL), normal glucose of 57 mg/dL (range: 40-70 mg/dL), and mild protein elevation to 54 mg/dL (range: 15-45 mg/dL). MRI of the lumbar spine showed enhancement of the cauda equina nerve roots on post-contrast images (Figure 1) concerning for an acute inflammatory process.

**Figure 2:**
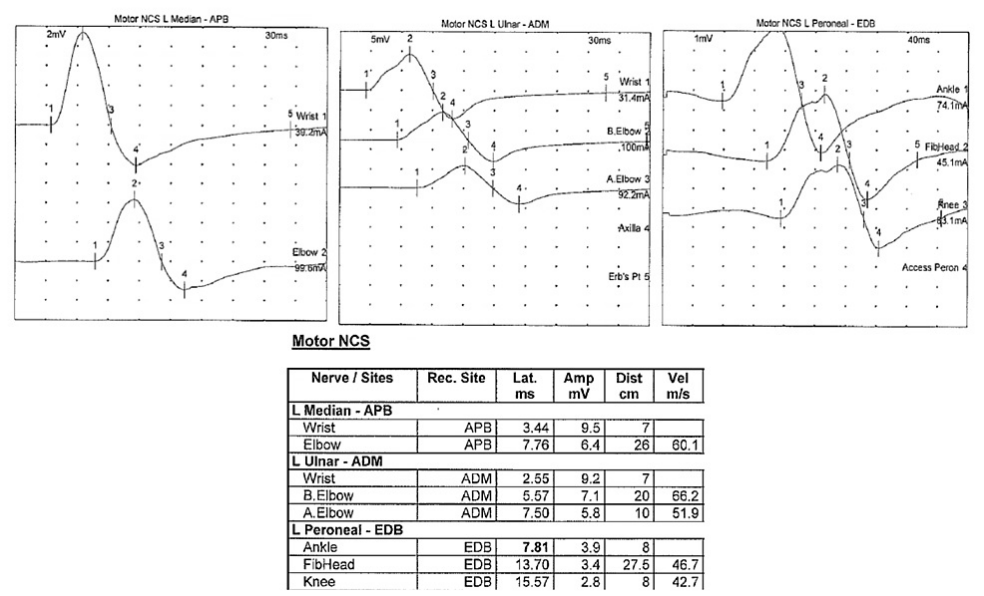
Motor nerve conduction study Prolonged distal motor latency in the left peroneal motor study with conduction block in the left median and ulnar nerves. APB: abductor pollicis brevis; ADM: abductor digiti minimi; EDB: extensor digitorum brevis; NCS: nerve conduction study.

**Figure 3:**
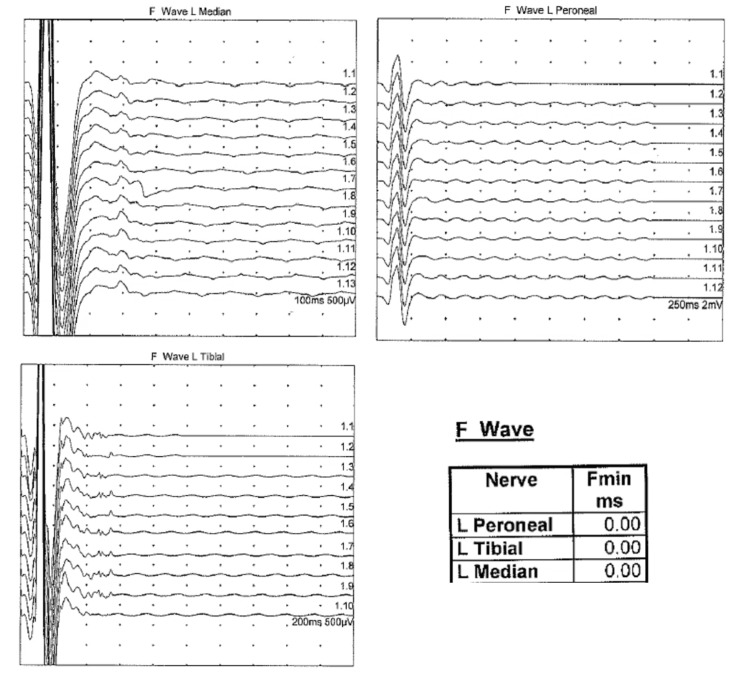
F-wave responses Absence of left peroneal, tibial, and median F-wave responses after repetitive supramaximal stimulations.

Electrophysiological studies were performed and showed prolonged distal motor latency in the left peroneal motor study with conduction block in the left median and ulnar nerves (Figure 2) as well as the absence of the left peroneal, tibial, and median F-wave responses (Figure 3). Needle electromyography (EMG) revealed fast-firing motor units with decreased recruitment. Those findings were suggestive of acute inflammatory demyelinating polyneuropathy (AIDP).

Given the clinical, radiographic, and neurodiagnostic concern for GBS, the patient was started on a five-day course of intravenous immunoglobulin (IVIG) (0.4 g/kg/day). On hospital day three, his unremarkable baseline respiratory parameters deteriorated (negative inspiratory force (NIF) decreased from -40 cm H2O to -28 cm H2O and vital capacity (VC) reduced from 2300 mL to 1300 mL), along with difficulty managing oral secretions for which he was intubated and transferred to the neurocritical care unit for respiratory support and further management.

**Figure 4:**
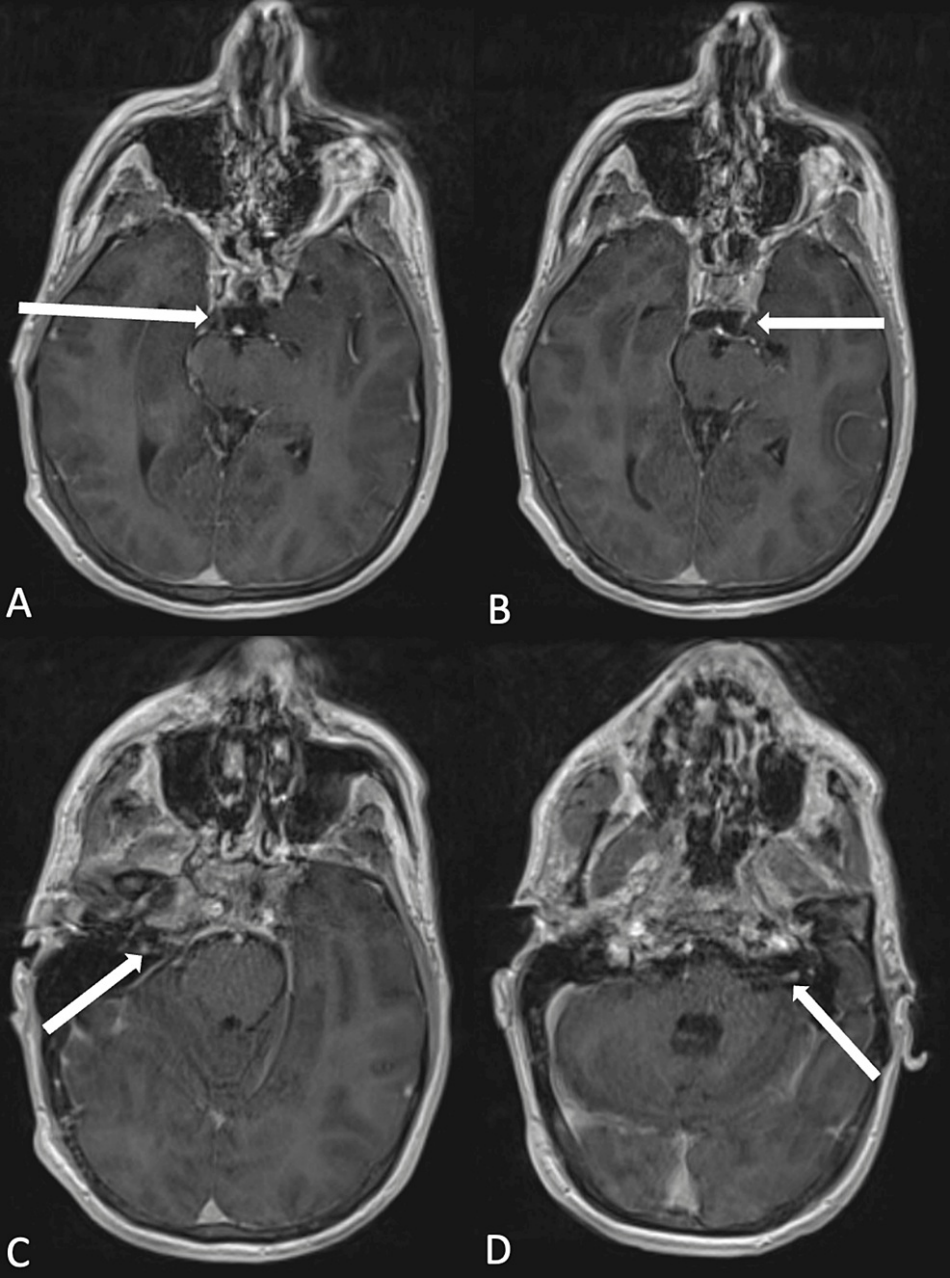
Axial brain MRI (contrast-enhanced T1-weighted) using cranial nerve protocol Multifocal abnormal cranial nerve enhancement involving cranial nerve III bilaterally (A and B) as well as cranial nerves VII/VIII in the internal auditory canals (C and D).

By hospital day eight, the patient’s condition continued to worsen despite having completed IVIG treatment. The physical exam revealed severe quadriparesis in bilateral upper and lower extremities (MRC scale of 1/5) with loss of sensation to light touch in all four extremities. His respiratory parameters continued to decline (VC = 1000 mL, NIF = -15 cm H2O), and subsequently, a tracheostomy was performed. Given the lack of improvement with IVIG for presumed GBS, Lyme serologies were tested and showed positive Lyme antibodies in serum and CSF. Additionally, western blot IgM for LD tested positive with all three diagnostic bands (p41, p39, p23) present. The patient was started on IV ceftriaxone and doxycycline for the concerns of neuroborreliosis. In the meantime, he developed dysconjugate gaze with bilateral ptosis. MRI brain with IV gadolinium showed increased multifocal abnormal cranial nerve (CN) enhancement involving CNs VII/VIII in the internal auditory canals and CN III bilaterally (Figure 4) suggestive of widespread leptomeningeal inflammation.

A week later, Lyme serologies were tested again and showed positive western blot IgM with two diagnostic bands present (p41, p23), in addition to positive western blot IgG, this time with five out of 10 diagnostic bands that are recommended by the Centers for Disease Control and Prevention (CDC) present (p66, p41, p39, p23, p18), confirming the diagnosis of LD.

Upon completion of a four-week course of antibiotics, the patient recovered sufficiently to be transferred to a long-term care facility. Two weeks later, the patient was weaned off ventilator support and discharged home. Eight months after the start of symptoms, the patient had recovered significantly. A follow-up exam in the neurology clinic revealed full upper extremities strength (MRC scale of 5/5) with only diminished lower extremities dorsiflexion and plantar flexion (MRC scale of 4/5) and mild paresthesia in his feet.

## Discussion

GBS is an immune-mediated polyradiculoneuropathy that classically presents with progressive (ascending) limb weakness and areflexia following an upper respiratory tract infection (URTI) or gastroenteritis. Several infections are associated with GBS, but *Campylobacter jejuni *is the most reported one [8]. In this case, the initial diagnosis of GBS was supported by the pattern of progressive ascending muscular weakness, paresthesia, and areflexia in combination with the albuminocytologic dissociation in CSF analysis one week after a URTI. It is important to note that CSF pleocytosis is a common feature of early LD, which was absent in this case [9]. Our working diagnosis of GBS was also supported by the nerve conduction study (NCS) that demonstrated a prolonged distal motor latency, conduction block, and absent F-wave response suggesting an underlying acute demyelinating pathology. Also, the enhancement of the cauda equina nerve roots with gadolinium on lumbosacral MRI images was concerning for an acute inflammatory process (83% sensitivity for acute GBS and presents in 95% of typical cases) [10].

The lack of clinical improvement after completing five days course of IVIG along with the patient history of “blotchy” abdominal rash prior to presentation in addition to the reported outdoor activities in wooded environments questioned the validity of GBS and raised the concern about an alternative diagnosis. Therefore, Lyme serologies were tested and showed positive Lyme antibodies in serum and CSF. Our patient had positive IgM western blot within 30 days of symptom onset with all three diagnostic bands present. This was followed by IgG seroconversion a week later with five out of 10 diagnostic bands present. The combination of the above-mentioned results confirmed the diagnosis of LD. Despite that our patient denied recalling a tick bite associated with his rash, a prospective study of 187 patients with *Borrelia burgdorferi* showed that only 26% of the patients recalled a tick bite, 46% noticed an erythema migrans and only 16% showed a skin rash (or residue of it) at the time of clinical evaluation [11]. In addition to the rash, our patient reported a history of fever and nonproductive cough a week before the presentation. These were likely signs of an early localized LD, as it typically includes nonspecific constitutional symptoms [4]. Other supportive features of Lyme neuroborreliosis (LNB) in our case include bilateral facial weakness and perioral numbness suggestive of cranial neuropathies, which is one of the most common manifestations of LNB (particularly facial palsy) [6]. This was shown on our patient's brain MRI as a multifocal abnormal cranial nerve enhancement suggestive of widespread leptomeningeal inflammation.

The pathophysiology of peripheral nerve injury in LNB is still unclear. Large studies have demonstrated multifocal lesions affecting both sensory and motor nerves acting as an axonal disease on NCS with relatively normal conduction velocities, only mild prolongation of late response latencies, and no evidence of conduction block [7]. Controversially, the electrodiagnostic evaluation of our patient showed a true demyelinating pathology. Only a few studies have suggested that demyelination may play a role in LNB [7].

Our patient had significant improvement two weeks after completion of the antibiotic course. Similar improvement of symmetric polyneuropathy following treatment for Lyme has been reported in rare instances. Tyagi et al. reported a 34-year-old woman with suspected GBS, which was refractory to a five-day course of IVIG and had significant improvement with subsequent antibiotic therapy once Lyme was diagnosed and treated [12]. Similarly, Owens et al. described a 30-year-old female who presented with suspected GBS that had some improvement with IVIG therapy. Continued motor dysfunction along with supportive Lyme studies prompted a 14-day course of ceftriaxone, after which the patient made a near full recovery [13]. Furthermore, similar studies often have simultaneous administration of IVIG with antibiotic therapy, which raises some doubt about the relationship between LNB and GBS symptoms [14]. In comparison, the patient described in our case continued to worsen following the IVIG course with significant improvement after completion of the antibiotic course. This pattern suggests that demyelination was secondary to LNB rather than a concurrent or coincidental pathology.

## Conclusions

We report a rare case of neuroborreliosis with clinical, radiographic, and neurodiagnostic findings mimicking GBS. Our high index of suspicion and comprehensive workup led to accurate diagnosis and appropriate management. LD can have diverse neurologic manifestations and should be considered in the differential diagnosis of GBS, especially when there is a history of outdoor activity. Early identification of this atypical pattern of presentation is the key to ensure quicker treatment and prevent disease progression.

## References

[1] Kugeler KJ, Schwartz AM, Delorey MJ, Mead PS, Hinckley AF (2021). Estimating the frequency of Lyme disease diagnoses, United States, 2010-2018. Emerg Infect Dis.

[2] Dumic I, Severnini E (2018). "Ticking bomb": the impact of climate change on the incidence of Lyme disease. Can J Infect Dis Med Microbiol.

[3] Schwartz AM, Hinckley AF, Mead PS, Hook SA, Kugeler KJ (2017). Surveillance for Lyme disease — United States, 2008-2015. MMWR Surveill Summ.

[4] Adkison H, Embers ME (2023). Lyme disease and the pursuit of a clinical cure. Front Med (Lausanne).

[5] Wormser GP, Wormser V (2016). Did Garin and Bujadoux actually report a case of Lyme radiculoneuritis?. Open Forum Infect Dis.

[6] Garcia-Monco JC, Benach JL (2019). Lyme neuroborreliosis: clinical outcomes, controversy, pathogenesis, and polymicrobial infections. Ann Neurol.

[7] Halperin JJ (2003). Lyme disease and the peripheral nervous system. Muscle Nerve.

[8] Shahrizaila N, Lehmann HC, Kuwabara S (2021). Guillain-Barré syndrome. Lancet.

[9] Schwenkenbecher P, Pul R, Wurster U (2017). Common and uncommon neurological manifestations of neuroborreliosis leading to hospitalization. BMC Infect Dis.

[10] Gorson KC, Ropper AH, Muriello MA, Blair R (1996). Prospective evaluation of MRI lumbosacral nerve root enhancement in acute Guillain-Barré syndrome. Neurology.

[11] Hansen K, Lebech AM (1992). The clinical and epidemiological profile of Lyme neuroborreliosis in Denmark 1985-1990. A prospective study of 187 patients with Borrelia burgdorferi specific intrathecal antibody production. Brain.

[12] Tyagi N, Maheswaran T, Wimalaratna S (2015). Neuroborreliosis: the Guillain-Barré mimicker. BMJ Case Rep.

[13] Owens J, Filatov A, Husain-Wilson S (2020). Guillain-Barre syndrome, neuroborreliosis, or both. Cureus.

[14] Schrestha K, Kadkhoda K (2022). Early Lyme disease-associated Guillain Barre syndrome: a case report. IDCases.

